# Severe Facial Herpes Vegetans and Viremia in *NFKB2*-Deficient Common Variable Immunodeficiency

**DOI:** 10.3389/fped.2019.00061

**Published:** 2019-03-19

**Authors:** Karyn Parsons, Sarah D. Cipriano, Lindsey B. Rosen, Sarah K. Browne, Jolan E. Walter, Bryan L. Stone, Susana Keeshin, Karin Chen

**Affiliations:** ^1^Department of Pediatrics, University of Utah School of Medicine, Salt Lake City, UT, United States; ^2^Department of Dermatology, University of Utah School of Medicine, Salt Lake City, UT, United States; ^3^Laboratory of Clinical Immunology and Microbiology, National Institute of Allergy and Infectious Diseases, National Institutes of Health, Bethesda, MD, United States; ^4^Center for Biologics Evaluation and Research, Food and Drug Administration, Silver Spring, MD, United States; ^5^Division of Allergy and Immunology, Department of Pediatrics, Johns Hopkins All Children's Hospital, St. Petersburg, FL, United States; ^6^Division of Allergy and Immunology, Department of Pediatrics, Morsani College of Medicine, University of South Florida, Tampa, FL, United States; ^7^Division of Allergy and Immunology, Department of Pediatrics, Massachusetts General Hospital for Children, Boston, MA, United States; ^8^Division of Pediatric Inpatient Medicine, Department of Pediatrics, University of Utah School of Medicine, Salt Lake City, UT, United States; ^9^Division of Pediatric Infectious Disease, Department of Pediatrics, University of Utah School of Medicine, Salt Lake City, UT, United States; ^10^Division of Allergy and Immunology, Department of Pediatrics, University of Utah School of Medicine, Salt Lake City, UT, United States

**Keywords:** NF-kappaB2, common variable immune deficiency (CVID), herpes vegetans, Herpes simplex virus (HSV), primary immunodeficiency (PIDD), anticytokine antibodies, hypogammaglobulinemia, pediatric primary immune deficiency

## Abstract

With the accessibility of next-generation sequencing modalities, an increasing number of primary immunodeficiency disorders (PIDDs) such as common variable immunodeficiency (CVID) have gained improved understanding of molecular pathogenesis and disease phenotype with the identification of a genetic etiology. We report a patient with early-onset CVID due to an autosomal dominant loss-of-function mutation in *NFKB2* who developed a severe herpes vegetans cutaneous infection as well as concurrent herpes simplex virus viremia. The case highlights features of CVID, unique aspects of NF-κB2 deficiency including susceptibility to herpesvirus infections, the detection of neutralizing anticytokine antibodies, and the complexity of medical management of patients with a PIDD that can be aided by a known genetic diagnosis.

## Introduction and Background

Common variable immunodeficiency (CVID; [MIM 607594]) is a primary immunodeficiency disorder characterized by hypogammaglobulinemia as well as poor humoral response to antigens or vaccines (1, 2). CVID is clinically and genetically heterogeneous, with onset of disease during childhood or adulthood. Patients typically present with a history of recurrent sinopulmonary infections, though some may have a predominance of autoimmune or immune dysregulatory features. Treatment universally includes gammaglobulin replacement. Less than 20% of CVID patients are identified to have familial cases or gene defects resulting in autosomal recessive or autosomal dominant forms of the disease (3, 4). In these forms of CVID, the genetic etiology provides a window into disease pathogenesis and potential disease complications that can develop over time (5). We present a case of a teenage patient with childhood-onset, autosomal dominant CVID caused by a heterozygous loss-of-function mutation in the gene *NFKB2* [NF-κB2; MIM 615577] who developed an unusually severe cutaneous herpes vegetans infection and herpes simplex virus (HSV) viremia requiring aggressive disease management.

## Case

**Figure 1:**
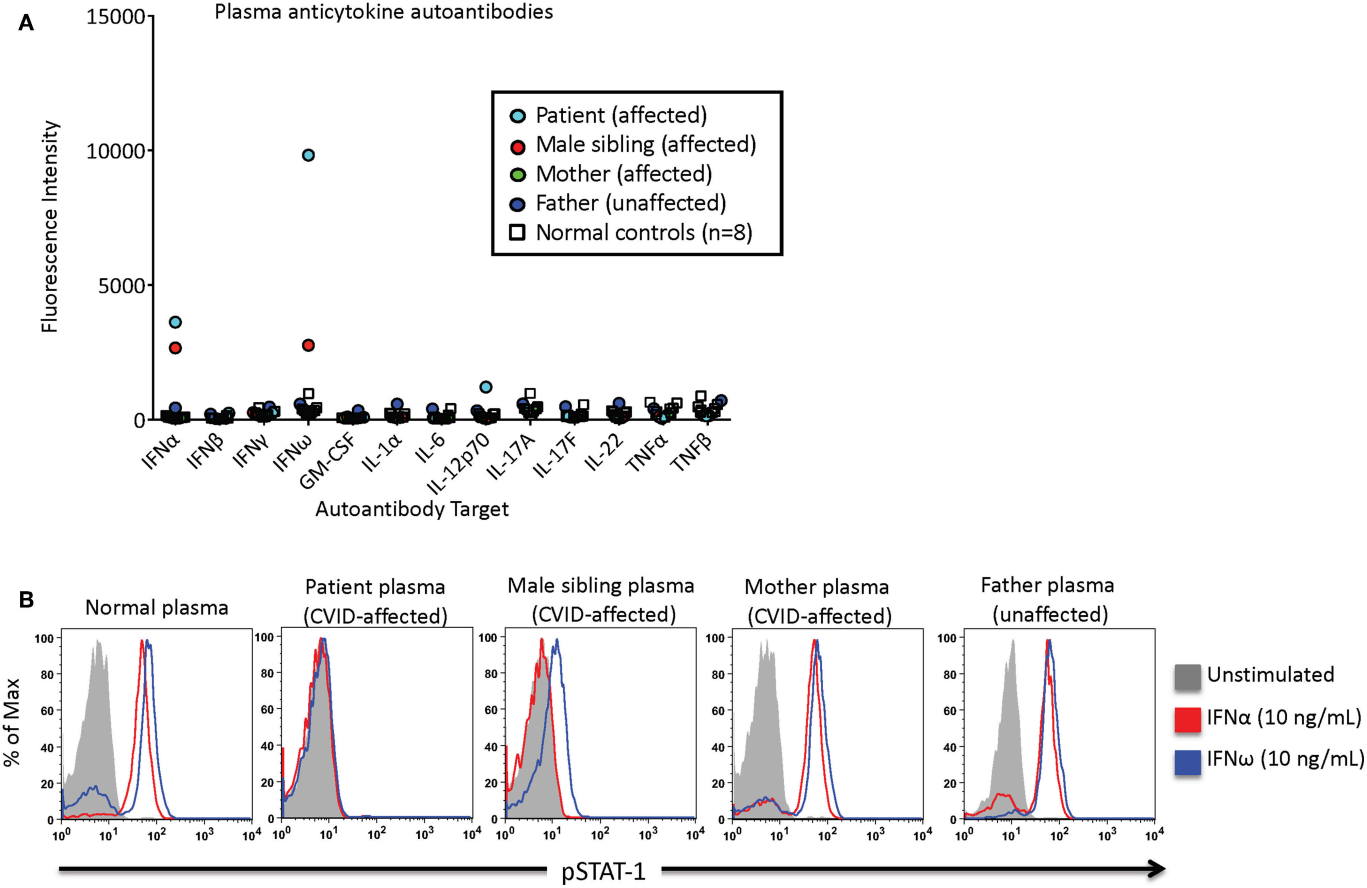
Patient, familial CVID-affected subjects, and control subject plasmas were screened for the presence of neutralizing anticytokine autoantibodies. **(A)** Anticytokine autoantibodies were quantified as a function of the fluorescence intensity using a Luminex-based assay. **(B)** IFN-α or IFN-ω induced phosphorylation of STAT1. Normal control peripheral blood mononuclear cells were incubated with subject or control plasma, and STAT1 phosphorylation was measured in unstimulated or stimulated conditions via flow cytometry.

An 18-year-old female was diagnosed with CVID at age six. As previously described in Chen et al. she, her mother, and younger brother were found to have a heterozygous pathogenic variant in *NFKB2* that results in an autosomal dominant form of CVID (6). She developed autoimmune manifestations, including acquired central adrenal insufficiency, alopecia universalis, vitiligo, and nail dystrophy. Her infectious history was significant for both bacterial and viral respiratory infections, recurrent herpes simplex of the lips and surrounding perioral skin, and chronic candidal onychomycosis. Adrenal insufficiency was treated with maintenance doses of hydrocortisone and stress dose adjustments during times of illness. Early-onset hypogammaglobulinemia required lifelong gammaglobulin replacement to prevent recurrent sinopulmonary infections. Recurrent herpes simplex infection was controlled with chronic suppressive oral valacyclovir therapy. A Luminex-based assay of the patient's plasma [1:100 dilution, as described by Ding et al. (7)] identified the presence of autoantibodies against interferon-α (IFN-α) and interferon-ω (IFN-ω) (Figure 1A). Next, patient plasma was incubated with control peripheral blood mononuclear cells and STAT1 phosphorylation was measured by flow cytometry after incubation in unstimulated or stimulated conditions as described by Burbelo et al. (8). The patient's plasma demonstrated neutralizing anti-IFN-α and IFN-ω antibodies which inhibited STAT1-phosphorylation in control cells, whereas control and parental plasma did not lead to inhibition (Figure 1B). The affected sibling's plasma also demonstrated neutralizing anti-IFN-α antibodies, and partial blockade of IFN-ω signaling.

At 16 years of age, the patient required intensive care hospitalization for decompensated septic shock of unknown etiology requiring vasopressor support. Prior to the hospitalization she had become non-compliant with medications, including gammaglobulin replacement. She developed acute renal injury, electrolyte disturbances, and adrenal crisis secondary to sepsis of unknown etiology which required stress dosing of steroids and electrolyte replacement. During this time, hypomagnesemia resulted in torsades de pointes and prolonged QT syndrome. Her persistent renal injury required chronic daily oral electrolyte replacement to prevent recurrence of arrhythmias.

Two years later, the patient presented to the emergency department with a month-long history of progressive, painful, vegetative facial lesions. The lesions initially developed periorally, similar to prior herpes simplex outbreaks. Despite increasing valacyclovir to treatment dosing, the lesions became purulent and continued to spread, involving the nose and right cheek. She was treated for presumed bacterial cellulitis with oral cefdinir. When a bacterial culture reportedly grew group B streptococcus, cefdinir was transitioned to oral clindamycin. Valacyclovir was discontinued as the lesions did not improve while on the medication. She reported tactile fevers and chills with continued progression of the vegetative lesions despite antibiotic treatment.

**Figure 2:**
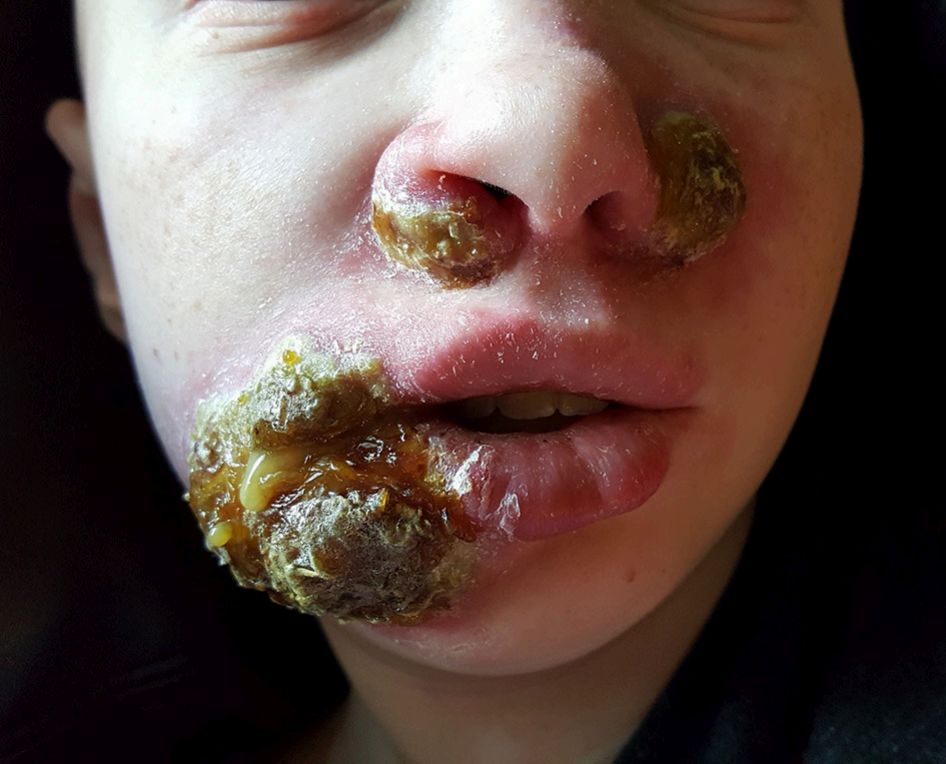
Herpes vegetans facial lesions at initial presentation.

**Figure 3:**
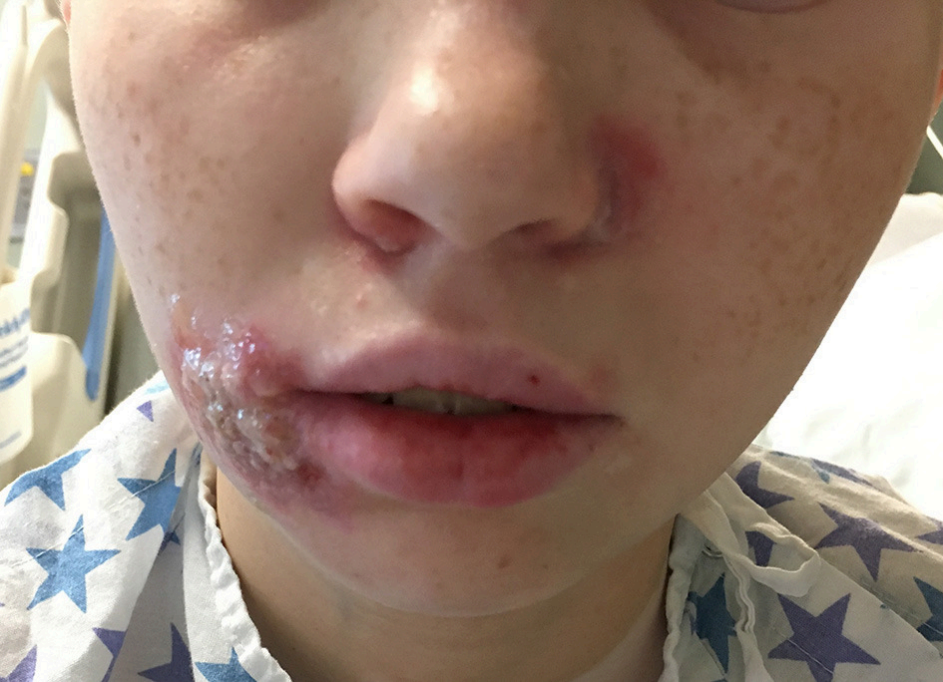
Herpes vegetans facial lesions after 10 days of intravenous foscarnet treatment.

**Figure 4:**
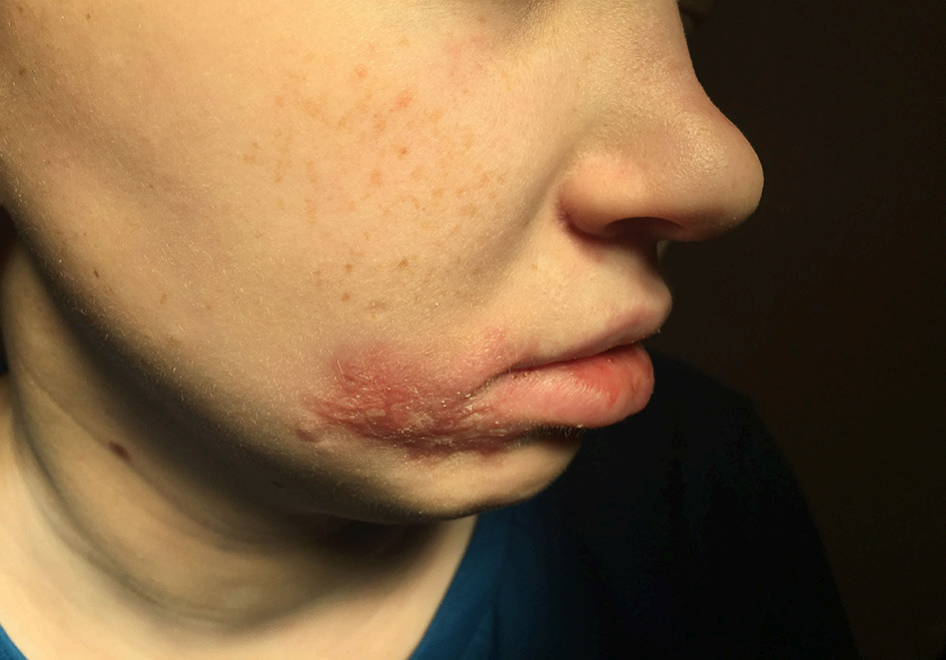
Facial lesions 2 months after presentation.

Upon hospitalization, laboratory evaluation identified leukocytosis (neutrophils 7,000 cells/μl; lymphocytes 7,900 cells/μl) and elevated C-reactive protein (11.6 mg/dL; normal range 0–1.5 mg/dL). Natural killer (NK) cell expression of CD107a was reduced, and NK cell functional assays demonstrated reduced cytotoxicity (Cincinnati Children's Diagnostic Immunology Laboratory). Her cutaneous exam revealed exophytic, thick, yellow-brown plaques involving the bilateral nares, right oral commissure, and adjacent medial cheek. The plaques were exudative and distorted the nasal architecture (Figure 2). A wound swab from the medial cheek was positive for HSV by polymerase chain reaction (PCR) assay. Other bacterial or viral infections often seen in immunodeficient patients, including varicella zoster and cytomegalovirus (CMV), were ruled out. The patient's systemic symptoms raised concern for HSV viremia, and a HSV peripheral blood PCR was positive. She did not have any neurological symptoms or meningeal signs; thus, HSV meningitis or encephalitis were not suspected. HSV type-1 was isolated from tissue culture of affected skin on the medial cheek; pathology demonstrated spongiosis and mixed granulomatous dermal infiltration with fat necrosis. Intravenous foscarnet therapy resulted in rapid improvement of her cutaneous eruption, including the associated pain, and systemic symptoms (Figure 3). Slower, continued resolution occurred over several weeks (Figure 4). The patient had recurrence of HSV vegetans and HSV viremia 3 months later. On this second occasion, early identification of HSV as the source of skin infection led to expedited hospitalization and early initiation of antiviral therapy. She had a significantly shorter hospital course with transition to oral valacyclovir at discharge when HSV culture demonstrated susceptibility to acyclovir.

## Discussion

Herpes vegetans is a rare, atypical cutaneous herpes simplex infection most commonly described in patients with acquired immunodeficiency due to HIV infection (9–11). Herpes vegetans has also been identified in other immunodeficient states, including CVID, congenital T-cell immunodeficiency, Good syndrome, malignancy, and those receiving immunosuppressive medications (12–15). Peripheral blood plasmacytoid dendritic cells from a patient with HIV-HSV coinfection had reduced IFN-α production, likely contributing to his severe, chronic HSV skin disease (16). Herpes vegetans can include exophytic, ulcerative, exudative, or verrucous lesions, and plaques. Tissue biopsy, direct immunofluorescence, and culture help confirm the diagnosis, guide the choice of antiviral therapy, and rule out other infectious etiologies or co-infections within the lesions. While acyclovir or valacyclovir are first-line therapies, acyclovir-resistant strains are often cultured from the lesions (11, 12) for which alternative antivirals including foscarnet, topical imiquimod, or intralesional cidofovir, should be considered (16–18). In our patient case, intravenous foscarnet treatment was initiated due to concerns for acyclovir resistance in the setting of apparent lack of response to oral valacyclovir, severity of infection, rapid progression of disease, and presence of HSV viremia. Foscarnet treatment should include careful monitoring for renal toxicity and electrolyte abnormalities including hypomagnesemia, hypocalcemia, and hypokalemia. Monitoring for foscarnet toxicity was particularly important in this case, given the patient's history of prior renal injury, long QT syndrome, and torsades des pointes.

In the setting of a primary immunodeficiency such as CVID, skin infections that are non-responsive to standard antibacterial therapies should raise concern for an atypical or opportunistic infection. In this particular case, herpes vegetans skin infection, herpes simplex virus (HSV) infection with antiviral resistance, and/or a superimposed fungal, bacterial, or atypical mycobacterial infection were all considered in the differential diagnosis. In the patient's family, all three CVID-affected family members had a history of recurrent HSV skin lesions. Not surprisingly, loss-of-function *NFKB2* mutations, leading to defective NF-κB2 protein function in the non-canonical NF-κB pathway, have been associated with susceptibility to viral infections, including HSV, varicella, herpes zoster, and molluscum contagiosum (6, 19–21). A *NFKB2* gain-of-function mutation resulting in a combined immunodeficiency phenotype has also been reported to have increased susceptibility to viral infections. One such patient developed CMV enteritis, severe Epstein-Barr virus (EBV) infection, warts, and herpes labialis (22). Thus, knowledge of the molecular or genetic pathogenesis of CVID or other PIDDs can be extremely important in specific disease screening and treatment decisions. In addition to infections typically seen in all patients with CVID, NF-κB2-deficient CVID patients are susceptible to viral infections and can develop adrenal insufficiency. The unique association of ACTH deficient-adrenal insufficiency in this genetic primary immunodeficiency disorder adds to the complexity of care in acute and chronic settings; stress dosing of steroids is critical in preventing life-threatening episodes of hypoglycemia and other complications associated with adrenal crisis (6, 20, 23).

At least two mechanisms may contribute to the increased susceptibility to viral infections in patients with *NFKB2* defects: (1) Defective NK cell cytotoxicity has been described in a patient with CVID-like disease due to *NFKB2* mutation (24); NK cells are critical in the cytotoxicity of virus-infected cells including herpes viruses (25), and (2) neutralizing anticytokine autoantibodies to interleukins and interferons have also been reported in an unrelated case of a CVID patient with a loss-function mutation in *NFKB2* who had severe recurrent bacterial and viral infections that improved after treatment with the anti-CD20 B-cell targeting monoclonal antibody, rituximab. Post-treatment, the patient had a significant reduction of autoantibody titers and no recurrence of invasive infections (21). In the current case report involving severe herpes vegetans and HSV viremia, abnormal NK cell function as well as neutralizing anti-IFN-ω and anti-IFN-α antibodies were identified, likely contributing to the recurrent and severe nature of the patient's HSV infection, and rituximab is being considered as a treatment option.

CVID is one of the most commonly treated primary immunodeficiencies, with an incidence of ~1:10,000 to 1:50,000 individuals (26–28). Criteria for diagnosis of CVID includes: (1) Significantly reduced immunoglobulin G serum levels as well as low levels of immunoglobulin A and/or immunoglobulin M, (2) poor or absent responses to antigens including immunizations, and (3) absence of any other well-defined primary or secondary hypogammaglobulinemic state (2). IgG serum levels vary significantly with age, and thus, care should be taken to use age-adjusted normal ranges in the evaluation of CVID. As some children may have transient hypogammaglobulinemia of infancy, a diagnosis of CVID is often not made until the child is at least 2–4 years of age. Though CVID is more often diagnosed in adulthood, one European cohort of more than 2,000 CVID patients found that onset of disease prior to 10 years of age occurred in 34% of the individuals. Thus, a diagnosis of CVID should be considered in any individual with recurrent sinopulmonary infections, or a constellation of recurrent or unusual infections along with autoimmune or immune dysregulatory disease manifestations.

NF-κB2, encoded by the gene *NFKB2*, is the principal protein of the non-canonical NF-κB pathway which has key roles in B-cell maturation, thymic development, and peripheral lymphoid organ development (29, 30). Loss-of-function mutations in *NFKB2* result in a highly penetrant form of childhood-onset hypogammaglobulinemia with recurrent infections, autoimmune features, and in many cases, endocrinopathy, or ectodermal features (31). The presence of autoimmunity and endocrinopathy in NF-κB2 deficiency could be explained by the importance of NF-κB2 in thymic expression of the *AIRE* gene, as demonstrated in murine models (32, 33). AIRE (autoimmune regulator) protein is required for development of central tolerance and elimination of self-reactive thymocytes. Reduced *AIRE* expression leads to the presence of circulating autoreactive T-cells, increasing susceptibility to autoimmune disease, and particularly in the endocrine system (34, 35). Endocrinopathies seen in *NFKB2* defects include ACTH-deficient adrenal insufficiency, hypothyroidism, and growth hormone deficiency. Ectodermal defects and autoimmune conditions include alopecia areata, trachyonychia, and vitiligo. Sinopulmonary infections are predominant, mucocutaneous candidiasis can occur, and as highlighted in this case, *NFKB2-*deficient patients can have increased susceptibility to herpesvirus infections. Recognition of primary immunodeficiency diseases including CVID and *NFKB2* defects allows for improved disease screening, targeted therapies, and the potential for research involving curative treatment.

## Data Availability

All datasets generated for this study are included in the manuscript.

## Ethics Statement

This study was carried out in accordance with the recommendations of the University of Utah Institutional Review Board with written informed consent from all subjects. All subjects gave written informed consent in accordance with the Declaration of Helsinki. Written informed consent was obtained from the participants for the publication of this case report with authorization to photograph and release images. The protocol was approved by the University of Utah Institutional Review Board.

## Author Contributions

KP co-wrote the initial draft of the manuscript and gathered data. SC co-wrote the manuscript, gathered data, and contributed to the analysis. LR and SB completed the experiments and analyzed the data. JW analyzed the data and assisted in study design. BS and SK gathered data. KC co-wrote the initial draft of the manuscript, gathered data, and contributed to concept, and design of the research. All authors contributed to manuscript revision, read, and approved the submitted version.

### Conflict of Interest Statement

KC receives grant support from NIH and Shire. The remaining authors declare that the research was conducted in the absence of any commercial or financial relationships that could be construed as a potential conflict of interest.
